# Decline of Humoral and Cellular Immune Responses Against SARS-CoV-2 6 Months After Full BNT162b2 Vaccination in Hospital Healthcare Workers

**DOI:** 10.3389/fimmu.2022.842912

**Published:** 2022-03-02

**Authors:** Benjamin Bonnet, Hélène Chabrolles, Christine Archimbaud, Amélie Brebion, Justine Cosme, Frédéric Dutheil, Céline Lambert, Maud Junda, Audrey Mirand, Amandine Ollier, Bruno Pereira, Christel Regagnon, Magali Vidal, Bertrand Evrard, Cécile Henquell

**Affiliations:** ^1^ Clermont-Ferrand University Hospital (CHU Clermont Ferrand), Immunology Department, Clermont-Ferrand, France; ^2^ Clermont Auvergne University, UMR UNH, ECREIN, Clermont-Ferrand, France; ^3^ Clermont-Ferrand University Hospital (CHU Clermont Ferrand), 3IHP, Virology Department, Clermont-Ferrand, France; ^4^ Clermont Auvergne University, CNRS UMR, LMGE, Clermont-Ferrand, France; ^5^ Clermont-Ferrand University Hospital (CHU Clermont Ferrand), Preventive and Occupational Medicine, Clermont-Ferrand, France; ^6^ Clermont Auvergne University, CNRS, LaPSCo Physiological and Psychosocial Stress, Clermont-Ferrand, France; ^7^ Clermont-Ferrand University Hospital (CHU Clermont Ferrand), Clinical Research and Innovation Direction (DRCI), Biostatistics Unit, Clermont-Ferrand, France; ^8^ Clermont-Ferrand University Hospital (CHU Clermont Ferrand) 3 IHP, Clinical Research and Innovation Direction, Clermont-Ferrand, France; ^9^ Clermont-Ferrand University Hospital (CHU Clermont Ferrand), 3 IHP, Infectious Diseases Department, Clermont-Ferrand, France

**Keywords:** B cell response, T cell response, interferon gamma (IFNγ), COVID-19, mRNA vaccine

## Abstract

Clinical trials and real-world evidence on COVID-19 vaccines have shown their effectiveness against severe disease and death but the durability of protection remains unknown. We analysed the humoral and T-cell immune responses in 110 healthcare workers (HCWs) vaccinated according to the manufacturer’s recommended schedule of dose 2 three weeks after dose 1 from a prospective on-going cohort in early 2021, 3 and 6 months after full vaccination with the BNT162b2 mRNA vaccine. Anti-RBD IgG titres were lower in HCWs over 60 years old 3 months after the second dose (p=0.03) and declined in all the subjects between 3 and 6 months with a median percentage change of -58.5%, irrespective of age and baseline comorbidities. Specific T-cell response measured by IGRA declined over time by at least 42% (median) in 91 HCWs and increased by 33% (median) in 17 others. Six HCWs had a negative T-cell response at 6 months. Ongoing follow-up should provide correlates of long-term protection according to the different immune response profiles observed. COVIDIM study was registered under the number NCT04896788 on clinicaltrials.gov.

## Introduction

The newly emergent severe acute respiratory syndrome coronavirus 2 (SARS-CoV-2) is responsible for the ongoing outbreak of viral pneumonia in humans called coronavirus disease 2019 (COVID-19). The disease, first identified in Wuhan, China, in late 2019, has spread very rapidly to become a global pandemic currently responsible for more than 4.5 million deaths (WHO). Vaccines against SARS-CoV-2, developed in a record time, are currently considered as the most promising approach to face this global health threat. The SARS-CoV-2 spike protein, including the receptor-binding domain (RBD), is the primary target of all currently available vaccines in Europe and the USA. Clinical trials and real-world data from vaccine deployment programmes have shown that full vaccination with COVID-19 vaccines is highly effective in preventing symptomatic infection, the need for hospitalisation, and death (1–5).

Although no correlate of protection has yet been established, the presence of neutralising antibodies due to prior natural infection has been associated with protection against subsequent SARS-CoV-2 infection (RT-PCR positive) and symptomatic disease (6, 7). Neutralisation is the gold standard assay for assessing the antibody response but requires a biosafety level 3 laboratory and highly-trained personnel. Immunoassays were highly correlated with neutralisation (8, 9) and the presence of anti-spike or anti-nucleocapsid antibodies was also associated with a reduced risk of SARS-CoV-2 reinfection in the ensuing 6 months (10). However, the rapid decline over time of the humoral immune response against natural SARS-CoV-2 infection is well documented (11–13). Data on cell-mediated immunity against SARS-CoV-2 are more subject to debate. Several reports suggested a decline in anti-spike specific CD4+ immune response after a few months (14, 15) whereas, in recent reports, cell memory responses were observed to be relatively stable over 8 months following natural infection with SARS-CoV-2 (16–19). Clinical factors related to medical history and acute infection could be associated with variability in T-cell responses over time (19).

One key question regarding COVID-19 vaccines is the duration of protection, particularly in the context of the emergence of variants of concern. Waning protection against SARS-CoV-2 infection up to 6 months after the second dose was recently reported in large national cohorts (20–23). A lower effectiveness of vaccines was also reported with the more transmissible variant Delta (4, 24, 25) with, however, a sustained protection against severe disease. These observations have raised concerns about the potential lack of durability of immunity to vaccination and reinforced the need for more immunogenicity data in different populations to evaluate current vaccine strategies. Persistence of serum antibodies is unlikely to be the sole determinant of long-lasting protection, and evidence supports a role of vaccine-induced cellular immune memory in reducing infection and disease (26). A few studies analysed the short-term cellular immune response following vaccination and showed that a maximum poly-specific cellular immune response against SARS-CoV-2 was elicited in a 3-week period immediately after BNT162b2 vaccination (27, 28) and started to decrease over one month (28). The long-term specific T-cell response following vaccination is still little documented except in few studies reporting data on a small cohort of patients at 6 months post-vaccination (14, 29). The technical complexity of T-cell response analysis limits studies to a small number of individuals by a few specialized laboratories. Thus, monitoring the magnitude and kinetics of humoral and cellular responses over time using commercial and standardized assays suitable for clinical laboratories and amenable to automation could be useful in large cohorts to gain fuller knowledge of the immunity elicited by COVID-19 vaccines.

We enrolled healthcare workers (HCWs) among a prospective longitudinal COVIDIM cohort of 300 volunteers from the University Hospital of Clermont-Ferrand, France, to assess immune response dynamics after vaccination against SARS-CoV-2. Sequential serum samples were collected 3 (M3), 6 (M6), 12, 18 and 24 months after the last dose of vaccine. Here, we analyse the sub-group of HCWs who had their M6 visit on August 31, 2021. They belonged to the first group vaccinated in France in early January 2021 with two doses 21 days apart of the BNT162b2 mRNA vaccine. Both antibodies against SARS-CoV-2 RBD and spike-specific T-cell response were evaluated using an interferon-gamma (IFN-γ) release assay (IGRA) 90 (+/- 15) and 180 (+/- 15) days after the second dose of vaccine.

## Materials and Methods

### COVIDIM Study Design and Participants

We conducted a prospective longitudinal cohort study at the University Hospital of Clermont-Ferrand, France. The main objective was to evaluate the durability of the humoral immune response to COVID-19 vaccination at 3, 6, 12, 18 and 24 months after the last dose of COVID-19 vaccine (one or two doses depending on prior infection or not). Secondary objectives included monitoring SARS CoV-2-specific T-cells immune responses using an *in vitro* IGRA. From May to September 2021, 300 volunteers were enrolled among 18- to 65-year-old hospital workers who had received vaccination against COVID-19 and who were in direct contact with patients or not. In accordance with the national strategy, vaccination began for >55-year-old HCWs in early January 2021 with the mRNA vaccine BNT162b2 developed by Pfizer/bioNtech. Vaccination was rapidly extended to younger personnel with the Astra-Zeneca (ChAdOx1 nCoV-19) vaccine and the Moderna mRNA vaccine, available in our hospital from 8 and 25 February 2021, respectively. Demographic data, risk factors defined as being associated with severe COVID-19 infection, prior COVID-19 infection or not, and vaccine details (name and injection dates) were collected. The study was approved by the Ile-de-France VIII ethics committee of France and registered on ClinicalTrials.gov (NCT04896788). Written informed consent was obtained from all participants before enrolment.

### Interim Analysis

On 31 August 2021, all participants who had attended the M6 follow-up visit after the second dose of Pfizer/bioNTech vaccine were included in an interim analysis. We recorded the kinetics of humoral immunity on the basis of serological criteria (anti-RBD antibody levels) and cellular immunity (measurement of IFN-γ released by antigen-specific T-cells after overnight stimulation with spike- specific peptides), 90 and 180 days (+/- 15 days) after the full vaccination.

### SARS-CoV-2 Antibody Immunoassays

SARS CoV-2 IgG levels were measured by an automated chemiluminescent microparticle immunoassay performed with the Abbott ARCHITECT instrument according to the manufacturer’s instructions. The Abbott SARS-CoV-2 IgG II Quant assay is designed for the quantitative detection of IgG antibodies to RBD of the SARS-CoV-2 spike protein (30). Anti-RBD response was defined as titre ≥50.0 arbitrary units (AU)/mL, or ≥7.1 binding antibody units (BAU)/mL as expressed in reference to the first WHO international standard for SARS-CoV-2 immunoglobulin (NIBSC 20-136), according to the manufacturer’s information.

Each serum collected at M3 was also tested with the Abbott SARS-CoV-2 anti-nucleocapsid protein IgG assay using the ARCHITECT instrument for screening prior unknown COVID-19 infection. According to the manufacturer’s instructions, the index values ≥1.4 are considered to be positive.

### SARS-CoV-2 Specific T-Cell Response: IFN-γ Whole Blood Assay

Vaccine-induced T-cell responses were assessed by a whole blood IGRA. According to the manufacturer’s information, the QuantiFERON (QFN) SARS CoV-2 Ag.1 tube contains CD4+ overlapping epitopes derived from the S1/Receptor-binding domain subunit of the spike protein, and the QFN SARS-CoV-2 Ag.2 tube contains CD4+ and CD8+ epitopes from the S1/RBD and S2 subunits. Blood was drawn directly into QFN tubes. Whole blood samples were incubated for 16 to 24 h at 37°C within 8 h of collection and then centrifuged and stored at 4°C until use. IFN-γ levels were quantified in international units (IU)/mL using an enzyme-linked immunosorbent assay performed according to the manufacturer’s instructions, with a 4-point standard curve. Results were reported as Ag.1 or Ag.2 without background signals from negative controls which had been subtracted from raw data.

In this research use only kit, the manufacturer did not provide any cut-off values to define a positive SARS CoV-2-specific T-cell response. Cut-off values of SARS-CoV-2 Ag.1 or SARS-CoV-2 Ag.2 for prediction of a positive cellular response were defined for the COVIDIM study by a receiver operating characteristics (ROC) curve analysis of the 110 vaccinated HCWs and 6 healthy controls using the pROC package in R software. The corresponding optimal cut‐off points were determined by Youden’s index using R software. The optimal cut-off of 0.015 IU/mL was defined for Ag.1 with 95.4% (95% confidence interval [CI], 90.7% to 99.1%) of sensitivity and 100% (95% CI, 100% to 100%) of specificity. For Ag.2, the sensitivity and specificity were 97.2% (95% CI, 93.5% to 100%) and 100% (95% CI, 100% to 100%), respectively, at a cut-off value of 0.016 IU/mL. The ROC curves for Ag.1 or Ag.2 are shown in **Supplemental Figure 1**.

Different groups of participants were identified according to the thresholds. Positive vaccinees were defined as participants with IFN-γ responses above the cut-off with both Ag.s. Vaccinees with at least an IFN-γ response above the threshold for one of two Ag.s were identified as partial responders. Participants with no IFN-γ response were identified as non-responders. Stronger response for Ag.2 than for Ag.1 was defined as IFN-γ for Ag.2/IFN-γ for Ag.1 ≥ 2 associated with a difference of response between the two Ag.s > 0.1 IU/mL. Participants with an increasing IFN-γ response between the two time points were identified as follows: IFN-γ increase between M3 and M6 > 0 IU/mL with both Ag.s, or IFN-γ increase for only one Ag with a ΔIFN-γ ≥ 0.09 IU/mL between the two time points.

### Statistical Methods

Statistical analysis was performed using Stata software (version 15; StataCorp, College Station, Texas, USA) and R software [version 3.5.3; R Foundation for Statistical Computing, Vienna, Austria]. All tests were two-sided, with a Type I error set at 0.05. No correction for multiple testing was applied in the analysis of secondary outcomes or subgroup analysis (31).

Categorical data were expressed as number of participants and associated percentages, and continuous data as mean ± standard deviation or median [interquartile range, IQR], according to statistical distribution.

The evolution of anti-RBD IgG, IFN-γ response with Ag.1, and IFN-γ response with Ag.2 between M3 and M6 was studied using linear mixed models, considering the time as a fixed effect and the participant as a random effect to model between- and within-subject variability. Logarithmic transformations were used when appropriate to achieve normality. The results were presented as effect size (ES) and 95% confidence interval (CI), and interpreted according to Cohen’s recommendations (32): 0.2 = small effect, 0.5 = medium effect and 0.8 = large effect. The factors associated with the variation of these criteria were also studied with linear mixed models but with the following fixed effects: time (M3 or M6), characteristics of the participants (e.g. sex), and their interaction. In addition, the factors associated with anti-RBD IgG, IFN-γ response with Ag.1, and IFN-γ response with Ag.2 at M3 on the one hand and at M6 on the other hand were studied with the Mann-Whitney test (all variables such as sex were binary).

To explore the relationships between humoral and T-cell responses after full vaccination with BNT162b2, Pearson’s correlation coefficients (noted Rho) were calculated. Comparison between independent groups were made with the Mann-Whitney test.

## Results

### Participant Characteristics

One hundred and ten participants from the COVIDIM cohort were included in this analysis. Their characteristics on enrolment are provided in **Table 1**. They had a mean age of 54 ± 8 years, 90 (81.8%) were female and 84 (76.4%) worked in direct contact with patients. All received two doses of the Pfizer/bioNTech mRNA vaccine 21 days apart and were fully vaccinated between 2 February and 12 March 2021. The mean time elapsed between their second vaccine injection and serum collection at M3 and M6 were 102 ± 5 and 175 ± 9 days, respectively. All but one of the 110 participants (99.1%) had no evidence of prior SARS-CoV-2 infection; the remaining subject (0.9%) had a history of proven infection assessed by RT-PCR swab 4 months before vaccination. Factors associated with severe COVID-19 outcome were identified in 22 (20.0%) HCWs, without change at the M6 time point. Obesity, defined as body mass index ≥30 kg/m^2^, was the most frequent comorbidity factor (13/22, 59.1%). On 31 August 2021, one participant experienced mild upper respiratory symptoms of COVID-19 and was confirmed positive by RT-PCR, 166 days after her last dose of vaccine. Whole genome sequencing identified a Delta variant (lineage B.1.617.2).

**Table 1:** Baseline characteristics of the healthcare workers vaccinated with the BNT162b2 mRNA vaccine.

Baseline characteristics	n=110
Female, n (%)	90 (81.8)
Age (years), mean (SD)	53.8 (7.6)
Age group, n (%)	
<60 years	86 (78.2)
≥60 years	24 (21.8)
Occupation, n (%)	
Contact with patients	84 (76.4)
No contact with patients	26 (23.6)
BMI (kg/m^2^), mean (SD)	24.8 (4.7)
BMI ≥30 kg/m^2^, n (%)	13 (11.8)
Comorbidities, n (%)	
No	88 (80.0)
One factor	15 (13.6)
Obesity	8
Hypertension	2
Respiratory disease	1
Diabetes	1
Immunosuppression	1*
Solid tumor	2
Two factors or more	7 (6.4)
Obesity + diabetes	1
Obesity + cardiovascular disease	1
Obesity + respiratory disease	1
Obesity + immunosuppression	1**
Respiratory disease + immunosuppression	1*
Diabetes + cardiovascular disease	1
Obesity + diabetes + cardiovascular disease	1

*Betamethasone regimen.

**Methotrexate and hydroxychloroquine regimen.

BMI, body mass index; SD, standard deviation.

### Humoral Response at M3 and M6 After Vaccination

**Figure 1:**
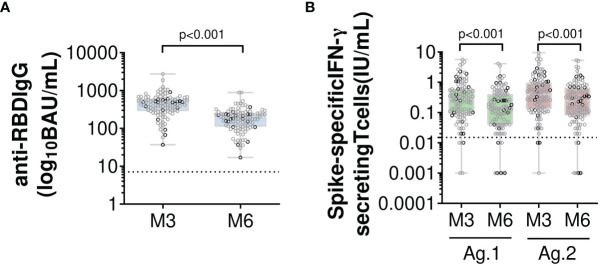
Humoral and specific T-cell responses in 110 healthcare workers 3 and 6 months after the second dose of the BNT162b2 mRNA vaccine. Evolution over time of **(A)** serum anti-RBD IgG antibodies and **(B)** IFN-γ secreting memory T-cells *via* an Interferon-Gamma Release Immunoassay that uses two mixes of SARS-CoV-2 spike protein (Ag.1 and Ag.2) selected to activate both CD4+ and CD8+ T-cells. Box and whiskers plot indicate median and interquartile range associated with min to max. All participants are indicated, and sex is discriminated by grey (women) or black (men) circle. Black dotted lines represent respective positive thresholds (p<0.001 *versus* M3, linear mixed-effects models). Number of cases for each time point (a) M3, 110; M6, 110; (b) M3, 108; M6, 110. BAU, binding antibody unit; IFN-γ, interferon gamma; IU, international unit; M3, 3 months post complete vaccination; M6, 6 months post complete vaccination; RBD, receptor-binding domain.

Antibody responses were assessed by enzyme-linked immunosorbent assays using the Abbott ARCHITECT instrument. IgG anti-SARS-CoV-2 nucleocapsid (N) was used for screening unknown infections that might have occurred in the previous few months before vaccination or between vaccination and the first time point of the study. Detection of anti-N antibodies was positive in three HCWs who did not report known SARS-CoV-2 infection, and negative in the one HCW with PCR-confirmed infection before vaccination. Titres of anti-RBD IgG were measured at M3 and M6. According to the manufacturer’s information, initial results expressed in arbitrary units (AU)/mL were converted to binding antibody units (BAU)/mL, which are traceable to the first WHO international standard for SARS-CoV-2 immunoglobulin. Anti-RBD antibodies were detected above the seropositivity threshold of 7.1 BAU/mL in all volunteers at M3 and M6. Median titres [IQR] were statistically lower at M3 in HCWs aged ≥60 years (343 [184; 468] BAU/mL *versus* 487 [304; 655] BAU/mL in HCWs <60, p=0.03), but were not significantly different at M6 (p=0.12) (**Table 2**). No significant differences were observed according to sex and baseline comorbidities at M3 and M6. Overall, the median titre [IQR] was significantly lower at M6 than at M3 (183 [112; 250] BAU/mL and 425 [288; 603] BAU/mL respectively, p<0.001, effect size (ES) (95% CI), -0.29 (-0.42 to -0.16)) (**Figure 1A**). Anti-RBD IgG titre declined in all participants, with a median [IQR] percentage change of -58.5% [-64.5; -52.6] between the two time points. Age, sex and comorbidity factors were not associated with the variation in anti-RBD IgG between M3 and M6 (data not shown).

**Table 2:** Effect of age, gender, body mass index and comorbidities on anti-RBD IgG and IFN-γ titers measured 3 and 6 months after vaccination in healthcare workers.

	M3						M6					
	Anti-RBD BAU/mL	*P*	IFN-y (Ag.1) IU/mL	*P*	IFN-y (Ag.2) IU/mL	*P*	Anti-RBD BAU/mL	*P*	IFN-y (Ag.1) IU/mL	*P*	IFN-y (Ag.2) IU/mL	*P*
**Age**												
<60 years *(n=86)*	487 [304; 655]	**0.03**	0.30 [0.09; 0.60]	0.71	0.49 [0.12; 0.97]	0.65	193 [118; 251]	0.12	0.16 [0.04; 0.40]	0.56	0.29 [0.08; 0.67]	0.46
≥60 years *(n=24)*	343 [184; 468]		0.25 [0.12; 0.43]		0.32 [0.15; 0.68]		146 [73; 234]		0.13 [0.05; 0.43]		0.15 [0.09; 0.52]	
**Gender**												
Female *(n=90)*	419 [287; 593]	0.31	0.25 [0.09; 0.60]	0.47	0.34 [0.13; 0.97]	0.84	181 [108; 241]	0.43	0.14 [0.04; 0.40]	0.51	0.24 [0.08; 0.67]	0.71
Male *(n=20)*	514 [352; 670]		0.35 [0.12; 0.58]		0.50 [0.13; 0.95]		209 [127; 277]		0.21 [0.07; 0.36]		0.31 [0.11; 0.61]	
**BMI**												
<30 kg/m^2^ *(n=95)*	425 [287; 620]	0.98	0.25 [0.09; 0.58]	0.27	0.34 [0.12; 0.97]	0.36	181 [108; 258]	0.95	0.14 [0.04; 0.40]	0.23	0.19 [0.08; 0.62]	0.14
≥30 kg/m^2^ *(n=13)*	397 [338; 556]		0.49 [0.13; 0.93]		0.58 [0.23; 0.92]		184 [159; 220]		0.21 [0.13; 0.53]		0.42 [0.30; 0.81]	
**Comorbidity**												
None *(n=88)*	423 [276; 595]	0.38	0.25 [0.09; 0.56]	0.17	0.34 [0.12; 0.95]	0.30	177 [107; 246]	0.23	0.14 [0.04; 0.39]	0.19	0.19 [0.08; 0.65]	0.22
At least one *(n=22)*	454 [338; 656]		0.46 [0.12; 1.25]		0.58 [0.18; 1.03]		206 [159; 252]		0.18 [0.10; 0.53]		0.36 [0.14; 0.81]	

Data are presented as median [interquartile range]. Statistical differences are shown in bold. BAU, binding antibody unit; BMI, body mass index; IFN-γ, interferon gamma; IU, international unit; M3, 3 months post full vaccination; M6, 6 months post complete vaccination; RBD, receptor-binding domain.

Anti-RBD IgG in the four HCWs with prior COVID-19 ranged from 37 to 1878 BAU/mL at M3 and from 17 to 888 BAU/mL at M6 (**Table 3**), with a decline over time similar to that measured in infection-naïve HCWs. During the follow-up period, one HCW, 60 years old, was symptomatic and was tested positive for SARS-CoV-2 by RT-PCR 166 days after her last dose of vaccine, the day before the M6 time point. Antibody titres were 118 BAU/mL at M3 and 53 BAU/mL at M6 and increased up to 4209 BAU/mL 17 days after positive RT-PCR testing.

**Table 3:** Anti-RBD IgG and IFN-γ titres 3 and 6 months after vaccination in healthcare workers according to prior infection or not.

	M3			M6			Time elapsed since infection
	Anti-RBD BAU/mL	IFN-γ (Ag.1) IU/mL	IFN-γ (Ag.2) IU/mL	Anti-RBD BAU/mL	IFN-γ (Ag.1) IU/mL	IFN-γ (Ag.2) IU/mL	
No prior COVID-19	425 [292; 592]	0.28 [0.10; 0.60]	0.39 [0.14; 0.94]	183 [113; 241]	0.14 [0.05; 0.40]	0.25 [0.09; 0.60]	not infected
Prior COVID-19							
known (RT-PCR positive)	67	0.02	0.03	37	0	0	4 months
unknown (anti-N positive at M3)	37	0.01	0.01	17	0	0.01	unknown
	1061	2.12	5.01	455	1.43	3.10	unknown
	1878	0.23	3.98	888	0.38	3.24	unknown
COVID-19 after vaccination	118	0.28	0.32	53	0.13	0.10	–

Data are presented as median [interquartile range]. BAU, binding antibody unit; IFN-γ, interferon gamma; IU, international unit; M3, 3 months post complete vaccination; M6, 6 months post full vaccination; N, nucleocapsid; RBD, receptor-binding domain; RT-PCR, reverse transcriptase-polymerase chain reaction.

### T-Cell Immune Response at M3 and M6 After Vaccination

SARS-CoV-2-specific memory T-cell responses were quantitatively analysed by IGRA, which is an *in-vitro* whole blood test measuring IFN-γ release by antigen-specific T-cells in response to stimulation by SARS-CoV-2 antigens (Ag.s). The QUANTIFeron test used two Qiagen proprietary mixes of SARS-CoV-2 spike protein (designated Ag.1 and Ag.2) selected to activate both CD4+ and CD8+ T cells. Receiver operating characteristics (ROC) curve analysis in vaccinated and non-vaccinated uninfected individuals determined optimal cut-off values of >0.015 IU IFN-γ/mL and >0.016 IU IFN-γ/mL for Ag.1 and Ag.2, respectively (**Supplemental Figure 1**). Also, the distribution of signals for the vaccinated subjects *vs*. healthy controls showed that vaccinated HCWs elicited a detectable IFN-γ response compared to healthy controls with an ineffective INF-γ response (p<0.001, ES=0.77 for Ag.1; p<0.001, ES=0.79 for Ag.2) (**Supplemental Figure 2**).

At M3 post-vaccination, two HCWs were excluded from the analysis because of an uninterpretable IFN-γ response due to the absence of stimulation of lymphocytes with mitogen. Two others, who were not immunocompromised, failed to develop a positive memory T-cell response, and 106 had a positive response combining Ag.1 and Ag.2 cut-off values. However, some differences in IFN-γ levels were observed according to the two Ag.s tested. Among the 106 responders, 17 showed a stronger response for Ag.2 (median [IQR] IFN-γ 0.95 [0.33; 1.63] IU/mL) than for Ag.1 (median [IQR] IFN-γ 0.23 [0.11; 0.49] IU/mL). In addition, three HCWs had a positive IFN-γ response after stimulation with Ag.2 only and conversely one had a positive IFN-γ response after stimulation with Ag.1 only. No difference in IFN-γ levels was observed at M3 according to sex, comorbidity factors or age (**Table 2**).

At M6, a positive SARS-CoV-2-specific memory T-cell response was observed in 104/110 (94.5%) HCWs. Of these, three had a detectable response only after stimulation with Ag.2 that in one was high (0.71 IU IFN-γ/mL). Overall, memory T-cell responses induced by the BNT162b2 vaccine decreased significantly over time, irrespective of age, gender or comorbidity factors (data not shown). Median [IQR] IFN-γ response dropped from 0.28 [0.09; 0.60] IU/mL and 0.39 [0.13; 0.96] IU/mL at M3 to 0.14 [0.04; 0.40] IU/mL and 0.25 [0.08; 0.67] IU/mL at M6 for Ag.1 and Ag.2, respectively (p<0.001 for both Ag.s, ES (95% CI): -0.29 (-0.42 to -0.12) for Ag.1 and -0.29 (-0.42 to -0.16) for Ag.2) (**Figure 1B**). This decline between M3 and M6 after vaccination was observed in 91/108 (84.3%) HCWs, with a median [IQR] percentage change of -50.0% [-66.9; -25.0] and -42.1% [-63.6; -27.9] between the two time points for Ag.1 and Ag.2, respectively. More surprisingly, memory T-cell response increased in 17/108 (15.7%) HCWs between M3 and M6. Specifically, 5 out of 17 had a very low increase over time (< 0.1 IU IFN-γ/mL for both Ag.s) (median [IQR] IFN-γ increase between M3 and M6: 0.03 [0.03; 0.04] for Ag.1 and 0.04 [0.02; 0.07] IU/mL for Ag.2), while the 12 others had a much higher increase (≥ 0.1 IU IFN-γ/mL for at least one Ag.) (median [IQR] IFN-γ increase between M3 and M6: 0.12 [0.07; 0.21] for Ag.1 and 0.19 [0.11; 0.33] IU/mL for Ag.2, respectively). In six HCWs, SARS-CoV-2-specific memory T-cell response was negative at M6. Of these, two were already non-responders at M3, and four had a low IFN-γ response at M3 that dropped below the cut-off values at M6 (median [IQR] IFN-γ response from 0.02 [0.02; 0.02] IU/mL and 0.03 [0.02; 0.03] IU/mL at M3 to 0.01 [0.00; 0.01] IU/mL and 0.00 [0.00; 0.01] IU/mL at M6 for Ag.1 and Ag.2, respectively). They were 52 to 59 years old, and none had a history of immunosuppression or immunosuppressive regimen.

In three out of the four HCWs with prior COVID-19, a positive IFN-γ response was observed at M3 with a decline at M6 similar to that measured in infection-naïve HCWs (**Table 3**). One of the three had a very low IFN-γ response at M3 (0.02 and 0.03 IU IFN-γ/mL for Ag.1 and Ag.2, respectively) and became negative at M6. In the HCW who experienced a SARS-CoV-2 infection after vaccination, the IFN-γ response measured 17 days after positive testing increased from 0.13 and 0.11 IU/mL at M6 to 1.49 and 1.36 IU/mL for Ag.1 and Ag.2, respectively.

### Correlation Between the Vaccine-Induced Humoral and T-Cell Responses

First, discordant results between humoral and T-cell responses were observed in several HCWs. At M3, two HCWs developed no IFN-γ response, and four had a weak and partial response for Ag.1 or Ag.2. All six developed anti-RBD antibodies, but at a lower titre than that of the others (median [IQR] anti-RDB IgG 196[73; 390] BAU/mL *versus* 433 [299; 629] BAU/mL, p=0.03). None was under immunosuppressive regimen. At M6, the same six HCWs had no detectable IFN-γ response with lower anti-RBD antibodies than other HCWs (median [IQR] anti-RDB IgG 83 [39; 142] BAU/mL *vs* 186 [114; 250] BAU/mL, p=0.04).

Second, 17 HCWs had an increased IFN-γ level at M6 compared to M3 with an overall median [IQR] percentage change of +42.5% [19.5; 67.0] for Ag.1 and +33.3% [27.1; 100.0] for Ag.2 while their anti-RBD titres declined with a median [IQR] percentage change similar to that of the others (-57.2% [-64.2; -50.2] *versus* -58.4% [-64.5; -53.0] respectively, p=0.56). Three of the 17 HCWs reported at-risk contact with acute COVID-19 patients but were tested negative by RT-PCR. One was exposed before vaccination and had positive anti-N antibodies while the other two, exposed after vaccination, were negative for anti-N antibodies.

**Figure 2:**
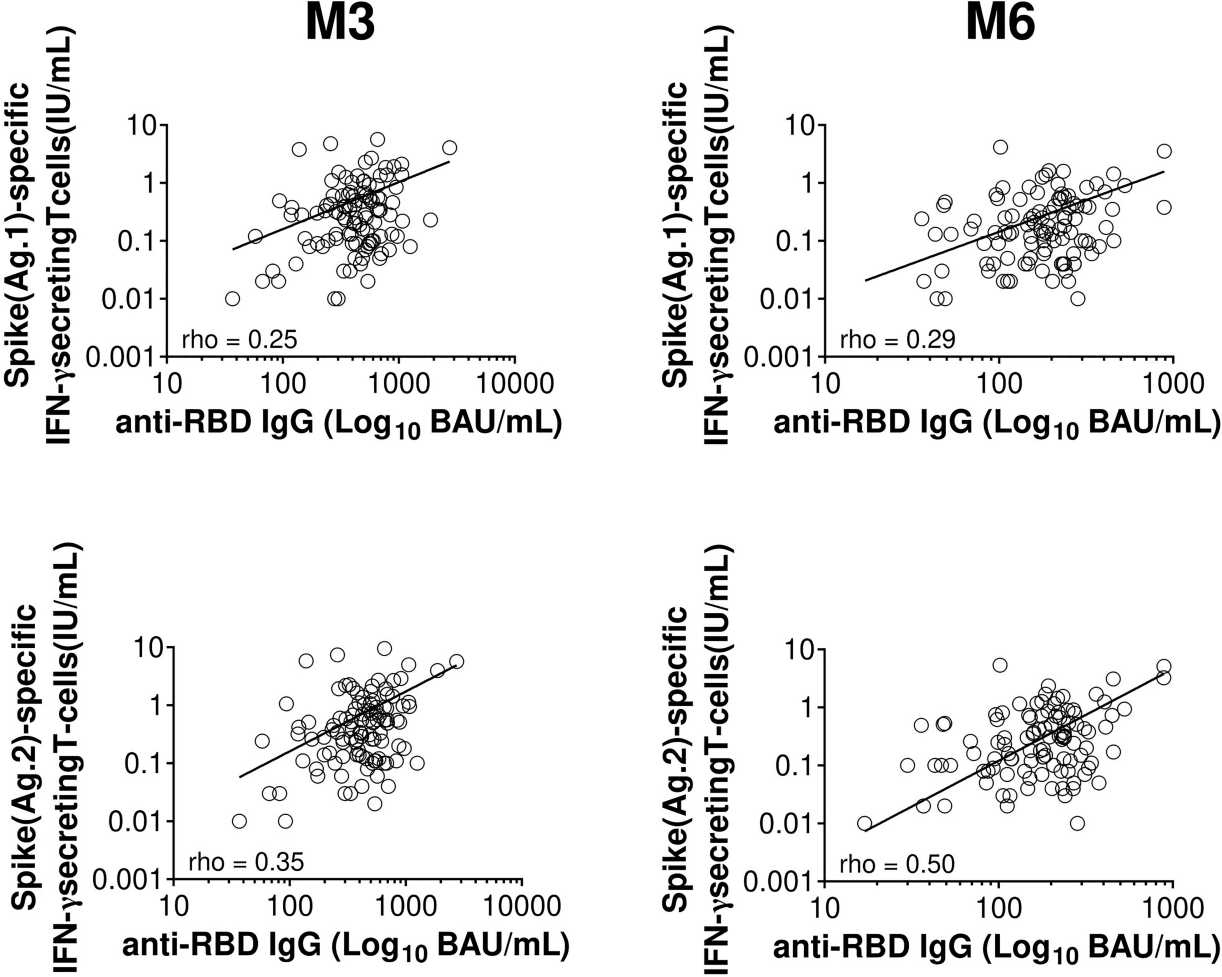
Correlation between humoral and T-cell responses 3 and 6 months after the second dose of BNT162b2 mRNA vaccine. Scatter plot of specific IFN-γ response and anti-RBD IgG over time following BNT162b2 vaccination (n=108 at M3 and n=110 at M6). The full line represents the best fit linear relationship of data. Pearson’s correlation coefficients are indicated (rho). BAU, binding-antibody unit; IFN-γ, interferon gamma; IU, international unit; M3, 3 months post complete vaccination; M6, 6 months post full vaccination; RBD, receptor-binding domain.

Despite these discordances, memory T-cell response to spike protein was correlated with anti-RBD IgG titres at M3 (Rho = 0.35, n=108) and at M6 to a much greater extent (Rho = 0.50, n=110) in our cohort (**Figure 2**). Similar results were found when the sub-group with increased T-cell response over time was excluded (Rho = 0.31, n=91 and Rho=0.44, n=91 at M3 and M6, respectively) (**Supplemental Figure 3**).

## Discussion

We monitored the immune response elicited by the Pfizer/bioNTech mRNA vaccine in accordance with the manufacturer’s recommended schedule in 110 HCWs for up to 6 months after the second dose injection. Anti-RBD IgG antibodies were detected at M3 in all participants, with a significantly lower titre in individuals aged ≥60 years. SARS CoV-2-specific T-cell responses were detected in 98.1% of participants. A notable inter-individual variation was observed for the antibody titres ranging from 37 to 2744 BAU/mL as for the IFN-γ response, ranging from 0.01 to 5.66 IU/mL and 0.01 to 9.52 IU/mL for Ag.1 and Ag.2, respectively. A rapid decline was evidenced between M3 and M6 post-vaccination. Median anti-RBD IgG dropped by 58.5% in all HCWs, while median T-cell specific response dropped by 50% and 42% for Ag.1 and Ag.2, respectively, in 84.3% of participants and became negative in four individuals. Conversely, T-cell response increased in 15.7% of HCWs between M3 and M6.

The decline in anti-RBD IgG 6 months after the second dose of BNT162b2 is consistent with recent reports in individuals who received the same mRNA vaccine (29, 33–35) or the mRNA-1273 vaccine (36). However, some differences should be noted. In our working population aged 22 to 65 years no antibody level below the positivity threshold was observed at M3 and M6 whereas in a population-based study in Israel it was found at the same time points in 5.8% and 16.1% of the vaccinated individuals (33). In contrast to the study of Levin et al. (34), lower titres were not associated in our study with male gender and immunosuppressive regimen. However, our results could be biased by the high percentage of women in the HCW population.

Six months after vaccination, one HCW experienced mild COVID-19 with a low anti-RBD IgG titre (53 BAU/mL), highly boosted by infection up to 4209 BAU/mL measured 17 days after positive RT-PCR. The relationship between the antibody titre and the risk of subsequent infection or disease (whether mild or severe) remains unknown. Recent studies proposed the first thresholds of protection based on antibody levels using a prediction model from immune data of clinical trials (37) or using the follow-up of a large French cohort of vaccinated and unvaccinated HCWs (38). In the French study, a titre ≥1700 BAU/mL provided full protection. In our cohort of HCWs, this titre was only reached in two individuals 3 months after the second dose of BNT162b2, and in none 6 months after. This clearly shows that reliable and standardized quantification of antibody response is a highly relevant question. In the present study, we chose a fully automated assay widely available for medical laboratories to measure anti-RBD IgG titres that has been proven to be highly correlated with neutralisation titres (8, 9). However, antibody levels should be used with caution. Several studies evidenced a good correlation between serological assays but showed that direct comparison of numerical results from different test systems was not possible, even when converted to binding antibody units (BAU) using the first WHO international standard (8, 39, 40). Further studies using different immunoassays and of different populations are thus required to define thresholds of anti-RBD IgG predictive of no, low or full protection after vaccination.

Our findings with regard to HCWs who experienced prior infection were intriguing. Previous studies suggested that one dose of mRNA vaccine induces similar or stronger immune responses in individuals with prior infection than those observed after the second dose in vaccinees without pre-existing immunity (41–43). Here, 4 out of the 110 HCWs had been previously infected (confirmed by positive RT-PCR or positive anti-N antibody detection) and received two doses of the BNT162b2 mRNA vaccine. They were expected to develop a strong and sustained immunity to vaccine. However, their immune response was remarkably variable. This sub-group of HCWs included the lowest titre (37 and 17 BAU/mL at M3 and M6, respectively) and the second highest titre (1878 and 888 BAU/mL) of antibodies in our cohort. The lowest titre, associated with a lack of detectable IFN-γ response, could be potentially explained by the interval of several months that had elapsed since the occurrence of an unknown asymptomatic infection revealed by the detection of anti-N antibodies. Low antibody titres (67 and 37 BAU/mL at M3 and M6, respectively) and negative IFN-γ response at M6 were found in another HCW who contracted mild COVID-19 4 months before vaccination. This finding suggests that immune priming of natural infection is not so effective or not long-lasting, at least in some immunocompetent individuals. It is possible that more HCWs experienced mild/asymptomatic COVID-19 before their enrolment in the study. Indeed, anti-N antibodies often became undetectable a few months after infection, particularly in case of mild symptoms, leading to underestimation of the true proportion of people with previous infection (44, 45).

Early immunogenicity studies demonstrate that mRNA vaccines induce CD4+ and CD8+ T cells responses (46) prior to the antibody responses (47, 48) with a maximum T-cell immune response observed in the month following the second dose of BNT162b2 (27, 28). Whether distinct T-cell phenotypes are involved in the long term T-cell evolution after COVID-19 vaccines remains to be solved. In natural infection, long-lasting SARS-CoV-2-specific memory T cells observed in convalescent COVID-19 patients displayed a phenotype of central memory CD4+ T cells (CD4+CCR7+CD45RA-) or stem cell-like memory CD4+ T cells (CD4+CCR7+CD45RA+) (18). Such an accurate phenotypic exploration of T cells is not possible using a commercial IGRA test. However, it allowed us to demonstrate for the first time, to the best of our knowledge, a decline in memory T-cell response against SARS CoV-2 in 84.3% of HCWs between 3 and 6 months after the second dose of the BNT162b2 mRNA vaccine. Most of the IFN-γ-producing CD4+ T lymphocytes involved in this assay had a phenotype of memory cells, with a major contribution of T effector memory cells (49). The short lifetime of these cells could explain the decline of IFN-γ responses over time observed in our study. However, this decline is consistent with the recent reports suggesting that spike-specific CD4+ T cells are downregulated at 3 and 6 months following vaccination (14, 28, 29, 50, 51) but contradict an interesting report suggesting that SARS-CoV-2–specific memory CD4+ T cells, based on the co-expression of CD200 and CD40L among CD45RA-, were relatively stable from 3 to 6 months after mRNA vaccination (52).

Another limit of this assay is the current absence of cut-off values defined by the manufacturer and validated by large studies. Other studies have evaluated T-cell responses with IGRA as a correlate of COVID-19 vaccination (53, 54) or as a diagnostic tool in natural infection (55), with different thresholds determined in each study. At the beginning of our study period, we had difficulty finding healthy unvaccinated donors who were naive for COVID19. We therefore set our threshold at a very small number of healthy controls (6 individuals), whose values nevertheless appear to be quite different from those of the vaccinated population. As for antibody response, the identification of predictors of no or low cellular immune response after vaccination as correlates of protection requires the definition of consensus cut-off values for the IFN-γ response measured by this assay.

Despite these limits, our results demonstrate the heterogeneity of the T-cell response elicited by the BNT162b2 mRNA vaccine in our cohort and identified several sub-groups of HCWs that would be interesting to follow up. Without evidence of SARS-CoV-2 infection, 17 (15.7%) vaccinees experienced an increasing memory T-cell response over time combined with a decreasing anti-RBD IgG titre. This could be explained by a previous exposure to seasonal coronaviruses leading to the generation of memory T cells specific for conserved epitopes (56, 57) and raises the question of the specificity of the SARS-CoV-2 IGRA, which needs to be tested in further large studies. This increasing memory T-cell response could also originate from the possible exposure to SARS-CoV-2 through at-risk contact with infected patients between M3 and M6, as documented for 2 of the 17 HCWs. Indeed, specific CD4+ and CD8+ memory T cells were found in close contacts tested negative by RT-PCR and seronegative (58), suggesting the possible development of SARS-CoV-2 T-cell immune response even in the absence of successful and detectable infection. IFN-γ response induced by Ag.2 was higher than that induced by Ag.1 in 17 other HCWs at M3 and M6 while their anti-RBD IgG response was similar to that of the others (median [IQR] anti-RBD IgG 487 [327; 674] *versus* 425 [288; 593] BAU/mL in others, p = 0.61, data not shown). This result suggests a preferential induction of a specific CD8+ T-cell response rather than a specific CD4+ T-cell response in these individuals. Functional spike-specific CD8+ T cells are elicited early after prime vaccination with the BNT162b2 mRNA vaccine (47), and in natural infection these effector cells are associated with a better outcome of COVID-19 and the development of durable protection (59). The long-term monitoring in the COVIDIM study of these two sub-groups of vaccinees, potentially more able to fight the virus, should teach us whether these T-response patterns are maintained and whether they are associated with better or longer protection. None of the vaccinees have so far been infected during the 6-month follow-up. Importantly, no HCW has had a negative humoral response associated with a specific T-cell response. Conversely, six HCWs had a SARS-CoV-2-specific IFN-γ response at M6 below our cut-off values. All had detectable anti-RBD IgG response at M3 and M6, and four of them had weak IFN-γ response at M3. An early decrease in T-cell response was recently observed by the sequential monitoring of patients over 30 days after the second dose of BNT162b2 (50). In this study, we report for the first time the cancellation of the T-cell response 6 months after full vaccination in a working population <60 years with no history of comorbidity or immunosuppression.

In conclusion, this study shows a high efficacy of the BNT162b2 mRNA vaccine in a cohort of HCWs 6 months after vaccination, with a single mild infection being recorded 166 days after the second dose of vaccine. The monitoring of humoral and T-cell responses using routinely available tests shed light on the high inter-individual variability of both anti-RBD Ig and IFN-γ responses after vaccination and their rapid decline over time. These results potentially raise the question of the usefulness of a third dose of vaccine. This regimen is already being applied in some countries for priority patients at high-risk for COVID-19-associated complications, namely the elderly, patients with chronic conditions, immunocompromised patients, and workers taking care of them. Several studies have shown a lower or defective humoral and T-cell immune response in these vulnerable patients after COVID-19 vaccination (60–62). An Israeli study in individuals aged ≥60 years who received a third dose of BNT162b2 5 months after the second one reported an average 10-fold increase in the titre of neutralizing antibodies with rapid and effective reduction of severe disease (63). However, such a booster strategy may not be necessary in all populations. Further longitudinal studies, including our own, in different populations vaccinated with different COVID-19 vaccines will help to determine whether the decline observed 6 months after the second dose will continue or not in the following months. They could also indicate how immune responses correlate with long-term protection against confirmed infection and severe disease, especially with the possible emergence of variants of concern that might threaten the efficacy of vaccines.

## Data Availability Statement

The raw data supporting the conclusions of this article will be made available by the authors, without undue reservation.

## Ethics Statement

The study was approved by the Ile-de-France VIII ethics committee of France and registered on ClinicalTrials.gov (NCT04896788). The patients/participants provided their written informed consent to participate in this study.

## Author Contributions

HC, BE, and BP planned the research. BB, CA, AB, JC, FD, MJ, AM, CR, MV, BE, and HC performed the enrolment of participants in the COVIDIM study. AO managed the study. BB and HC analysed the data. CL and BB realized the statistic tests. BB, BE, and HC wrote the paper. All authors contributed to the article and approved the submitted version.

## Funding

The COVIDIM study is funded by the Clinical Research and Innovation Direction of the Clermont-Ferrand University hospital.

## Conflict of Interest

The authors declare that the research was conducted in the absence of any commercial or financial relationships that could be construed as a potential conflict of interest.

## Publisher’s Note

All claims expressed in this article are solely those of the authors and do not necessarily represent those of their affiliated organizations, or those of the publisher, the editors and the reviewers. Any product that may be evaluated in this article, or claim that may be made by its manufacturer, is not guaranteed or endorsed by the publisher.
