# Supplementary material for: Decline of Humoral and Cellular Immune Responses Against SARS-CoV-2 6 Months After Full BNT162b2 Vaccination in Hospital Healthcare Workers
Source: Front Immunol. 2022 Mar 2;13:842912. doi: 10.3389/fimmu.2022.842912 (PMC8926062; doi:10.3389/fimmu.2022.842912)
Supplement: Supplementary file 1 [file DataSheet_1.docx]

Supplementary Material

## Supplementary Figures

**Supplemental Figure 1. Receiver operating characteristic (ROC) curves predicting SARS-CoV-2-specific T-cell response in 110 vaccinated healthcare workers**

Receiver operating characteristic (ROC) curves of IFN-γ secreting memory T-cells via an Interferon-Gamma Release Immunoassay (IGRA) that uses two mixes of SARS-CoV-2 spike (S) protein **(a)** S**-**Ag.1 and **(b)** S**-**Ag.2, obtained 3 months after the second dose of the BNT162b2 mRNA vaccine in 110 healthcare workers. Area under the ROC (AUC) curve with 95% confidence interval is indicated for each parameter.

**Supplemental Figure 2. SARS-CoV-2 QuantiFERON test is able to identify T-cell mediated responses in BNT162b2 vaccinated subjects**

Scatter plot of specific IFN-γ response induced by **(a)** Ag.1 or **(b)** Ag.2 showing the distribution of signals for the vaccinated subjects (n=110) vs healthy controls (n=6). Comparison between independent groups was made with the Mann-Whitney test and the results were presented as effect size and interpreted according to Cohen’s recommendations. Two steps were used to calculate effect-size: first, eta squared (η2) from Mann- Mann-Whitney-U test value was estimated, then a transformation in effect size was applied. IFN-γ: interferon gamma; IU: international unit.

**Supplemental Figure 3. Correlation between humoral and T-cell responses 3 and 6 months after the second dose of BNT162b2 mRNA vaccine**

Scatter plot of specific IFN-γ response induced by Ag.1 or Ag.2 and anti-RBD IgG in 91 (M3) and 91 (M6) participants with a concordant evolution of T- and B-cell responses over time following BnT162b2 vaccination. The full line represents the best fit linear relationship of data. Pearson’s correlation coefficients are indicated (rho). BAU: binding antibody unit; IFN-γ: interferon gamma; IU: international unit; M3: 3 months post full vaccination; M6: 6 months post full vaccination; RBD: receptor-binding domain.
